# Supplementary material for: 7S,15R-Dihydroxy-16S,17S-Epoxy-Docosapentaenoic Acid, a Novel DHA Epoxy Derivative, Inhibits Colorectal Cancer Stemness through Repolarization of Tumor-Associated Macrophage Functions and the ROS/STAT3 Signaling Pathway
Source: Antioxidants (Basel). 2021 Sep 14;10(9):1459. doi: 10.3390/antiox10091459 (PMC8470250; doi:10.3390/antiox10091459)
Supplement: Supplementary file 1 [file antioxidants-10-01459-s001.zip › antioxidants-1372403-supplementary.pdf]

Table S1. Specific primers for RT-qPCR analysis of human target and control (β-actin) genes

Genes	Accession Number	Primers
CD209	NM_001144895.2	Forward: 5'-CCA GGA TGG TCT CGA TCT CT-3'
		Reverse: 5'-CCA GGA TGG TCT CGA TCT CT-3'
CD163	NM_203416.4	Forward: 5'-CGTTCACTCTCAAGTCATCTGCG-3'
		Reverse: 5'-TAA GCT GGC AAA GAA CA-3'
CD206	NM_001009567.1	Forward: 5′-ACCTCACAAGTATCCACACCATC -3′
		Reverse: 5′-CTTTCATCACCACACAATCCTC -3′
MMP2	NM_001144893.2	Forward: 5′-ACC GCG ACA AGA AGT ATG GC-3′
		Reverse: 5′-CCA CTT GCG GTC ATC GT-3′
MMP9	NM_004994.3	Forward: 5′-CGA TGA CGA GTT GTG GTC CC-3′
		Reverse: 5′-TCG TAG TTG GCC GTG GTA CT3′
Trem2	NM_018965.4	Forward: 5′-TTG CCC CTA TGA CTC CAT GA -3′
		Reverse: 5′-CGC AGC GTA ATG GTG AGA GT -3′
VEGF	NM_001025366.3	Forward: 5′-ATG GCA GAA GGA GGG CA-3′
		Reverse: 5′-ATC GCA TCA GGG GCA CAC AG-3′
E-cadherin	NM_004360.5	Forward: 5'-GCC TCC TGA AAA GAG AGT GGA AG-3'
		Reverse: 5'-TGG CAG TGT CTC TCC AAA TCC G-3'
N-cadherin	NM_001792.5	Forward: 5'-CCTCCAGAGTTTACTGCCATGAC-3'
		Reverse: 5'-GTAGGATCTCCGCCACTGATTC-3'
Vimentin	NM_003380.5	Forward: 5'-AGGCAAAGCAGGAGTCCACTGA-3'
		Reverse: 5'-ATCTGGCGTTCCAGGGACTCAT-3'
β-actin	NM_001100.4	Forward: 5′-TGTTACCAACCTGGGACGACA-3′
		Reverse: 5′-GGGGTGTTGAAGGTCTCAAA-3′
